# Marcus-type driving force correlations reveal the mechanism of proton-coupled electron transfer for phenols and [Ru(bpy)_3_]^3+^ in water at low pH†

**DOI:** 10.1039/c6sc00597g

**Published:** 2016-04-01

**Authors:** Janne Soetbeer, Prateek Dongare, Leif Hammarström

**Affiliations:** a Department of Chemistry – Ångström Laboratory, Uppsala University Box 523, SE-751 20 Uppsala Sweden leif.hammarstrom@kemi.uu.se prateek.dongare@kemi.uu.se

## Abstract

Proton-coupled electron transfer (PCET) from tyrosine and other phenol derivatives in water is an important elementary reaction in chemistry and biology. We examined PCET between a series of phenol derivatives and photogenerated [Ru(bpy)_3_]^3+^ in low pH (≤4) water using the laser flash-quench technique. From an analysis of the kinetic data using a Marcus-type free energy relationship, we propose that our model system follows a stepwise electron transfer-proton transfer (ETPT) pathway with a pH independent rate constant at low pH in water. This is in contrast to the concerted or proton-first (PTET) mechanisms that often dominate at higher pH and/or with buffers as primary proton acceptors. The stepwise mechanism remains competitive despite a significant change in the p*K*_a_ and redox potential of the phenols which leads to a span of rate constants from 1 × 10^5^ to 2 × 10^9^ M^−1^ s^−1^. These results support our previous studies which revealed separate mechanistic regions for PCET reactions and also assigned phenol oxidation by [Ru(bpy)_3_]^3+^ at low pH to a stepwise PCET mechanism.

## Abstract 2

We examined PCET between a series of phenol derivatives and photogenerated [Ru(bpy)_3_]^3+^ in low pH (≤4) water using the laser flash-quench technique.

## Graphical abstract

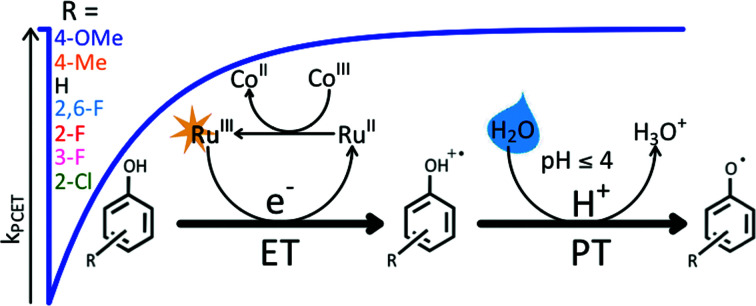

## Introduction

Proton-coupled electron transfer (PCET) reactions are ubiquitous in chemistry and biology. PCET is a term given to a wide array of reactions where proton transfer accompanies electron transfer.^1–5^ Various mechanistic regimes such as sequential electron–proton transfer (ETPT or PTET), concerted electron–proton transfer (CEP) and hydrogen atom transfer (HAT) reactions fall under the umbrella of PCET reactions.^5,6^ In many cases the step-wise and concerted mechanisms compete with each other, as has been found in model compounds as well as complex systems (polypeptides, enzymes) and therefore the ability to distinguish between the mechanisms is of prime importance. However, the intermediate species of the step-wise reactions are usually very short-lived and cannot be directly detected. In the majority of cases, thermodynamic-kinetic arguments are used to judge whether a step-wise mechanism is possible, *i.e.* if observed rate constants or activation energies are consistent with expected differences in *E*^0^ or p*K*_a_ between the reagents. Also, high H/D kinetic isotope effects (KIEs) are used to support assignment to a CEP reaction, but the value is often moderate, KIE ≈ 2.^7–13^ More rarely, rate constants *vs.* driving force correlations are used to support a mechanistic assignment. In one example, direct measurements of all the limiting ET, PT and CEP rate constants were made for the PCET oxidation of [W(Cp)(CO)_3_H] to conclude that CEP is the operating mechanism when both the oxidants and the bases are weak.^14^

Tyrosine and other phenol derivatives constitute the most studied class of compounds for PCET reactions, in synthetic systems as well as in proteins. The role of H_2_O in PCET reactions of phenolic compounds is topical with reference to natural photosynthetic membranes and other protein systems. Therefore the mechanism of PCET in water at different pH ranges remains an active area of intense investigation and debate.^10,12,15–29,48^ However, in aqueous systems it is often even more difficult to distinguish between stepwise and concerted reactions. Differences in reactant p*K*_a_ and *E*^0^ values are often less extreme than in organic solvents, and observed H/D KIEs may be affected by solvent KIEs of the H_2_O/D_2_O exchange.

A prototypical PCET reaction is the oxidation of phenol by [Ru(bpy)_3_]^3+^ derivatives with water as the primary proton acceptor. In neutral and alkaline solution the mechanism has been assigned to a CEP reaction, or a PTET reaction involving OH^−^.^10,15,17,30^ In some literature reports the pH-independent PCET mechanism at low pH (2–4), when OH^−^ was not the primary acceptor, was assigned to a CEP reaction based on the kinetic isotope effect KIE = 2.^15,17^ Irebo *et al.*^10^ instead assigned it to an ETPT reaction, as a pathway with different kinetic characteristics (*e.g.* KIE = 3.0) that dominated at neutral pH was already assigned to a CEP reaction; however, they did not exclude a CEP mechanism also at low pH. It should be noted that neither of these two mechanisms could be excluded based on thermodynamic limits, as discussed above for organic solvents. Also, while KIEs around 2 are often taken as evidence for a CEP reaction, even pure ET reactions may show a significant solvent KIE when H_2_O and D_2_O are compared.^10,31–33^

**Chart 1:**
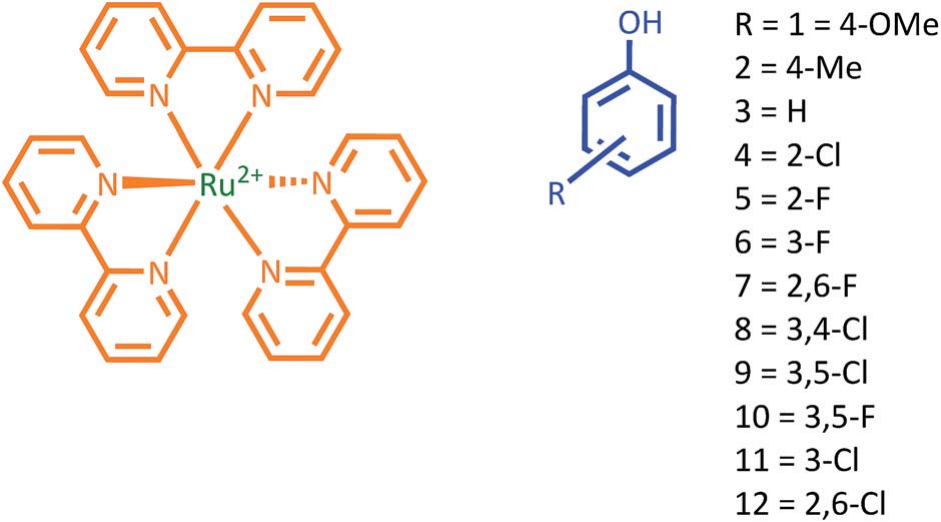
Structural formulae of [Ru(bpy)_3_]^2+^ and phenol derivatives. Phenols 8–12 were included only in the electrochemical analysis (see ESI†).

In order to investigate the PCET mechanism for this important case study reaction at low pH, we employed a series of phenols with varying redox potentials and p*K*_a_ values (Chart 1 and Table S3†), which were oxidized by laser-flash generated [Ru(bpy)_3_]^3+^. We investigated the correlation of experimental rates of phenol oxidation and the ones predicted for the CEP and stepwise mechanisms. Similar correlations were previously established by Mayer *et al.*^34^ to test PCET mechanisms in phenolic systems with organic bases.^3,35–37^ Reports have appeared where oxidation of phenol derivatives in organic solvents by [Ru(2,2′-bipyrazine)_3_]^2+^ has been studied.^38,39^ However, this is the first example of this method for deducing a PCET mechanism where water is acting as the proton acceptor.

## Results and discussion

**Fig. 1:**
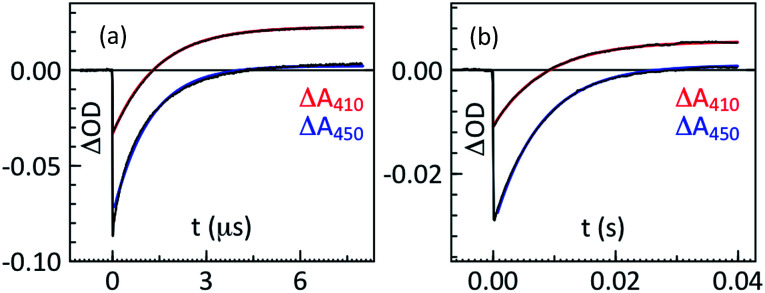
Representative kinetic traces at 450 and 410 nm for the oxidation of (a) phenol 1 (4-MeO) and (b) phenol 7 (2,6-F). The remaining traces are shown in the ESI (Fig. S1†).

The PCET reaction was studied using the ‘flash-quench’ method. The reaction was initiated by irradiation of [Ru(bpy)_3_]^2+^ at 460 nm using a 10 ns laser flash in the presence of an electron acceptor, [Co(NH_3_)_5_Cl]Cl_2_ or methylviologen (MV^2+^), which resulted in generation of a photooxidized [Ru(bpy)_3_]^3+^ species on a 100 ns time scale. The [Ru(bpy)_3_]^3+^ then oxidized the phenol on a much longer time scale, leading to a recovery of the [Ru(bpy)_3_]^2+^ bleach at around 450 nm and phenoxyl radical absorption at around 410 nm (Fig. 1).

The resulting transient absorption trace was subjected to a single exponential fit to extract the pseudo-first order rate constants for oxidation of phenol (*k*_obs_). To nullify the effect of recombination between MV^+^ and [Ru(bpy)_3_]^3+^, [Co(NH_3_)_5_Cl]^3+^ was used as a sacrificial acceptor instead of MV^2+^, which ensured that even the slowest PCET rates could be observed. To avoid interference from irreversible phenol degradation, phenol was added in great excess of [Ru(bpy)_3_]^2+^ and fresh, deoxygenated solutions which were protected from ambient light were used. Separate experiments were performed for phenols with faster rates (*i.e.*1–3) using the reversible acceptor MV^2+^ to ensure that the PCET rates are independent of the choice of external oxidant. At 450 nm and pH 2 the rate constants of 1.94 × 10^9^ M^−1^ s^−1^ and 3.69 × 10^5^ M^−1^ s^−1^ were obtained for phenols 1 and 3, respectively. This rules out any interference from by-products of the cobalt complex as rate constants of 1.9 × 10^9^ M^−1^ s^−1^ and 3.8 × 10^5^ M^−1^ s^−1^, respectively, were obtained when [Co(NH_3_)_5_Cl]Cl_2_ was used as an external electron acceptor.

**Fig. 2:**
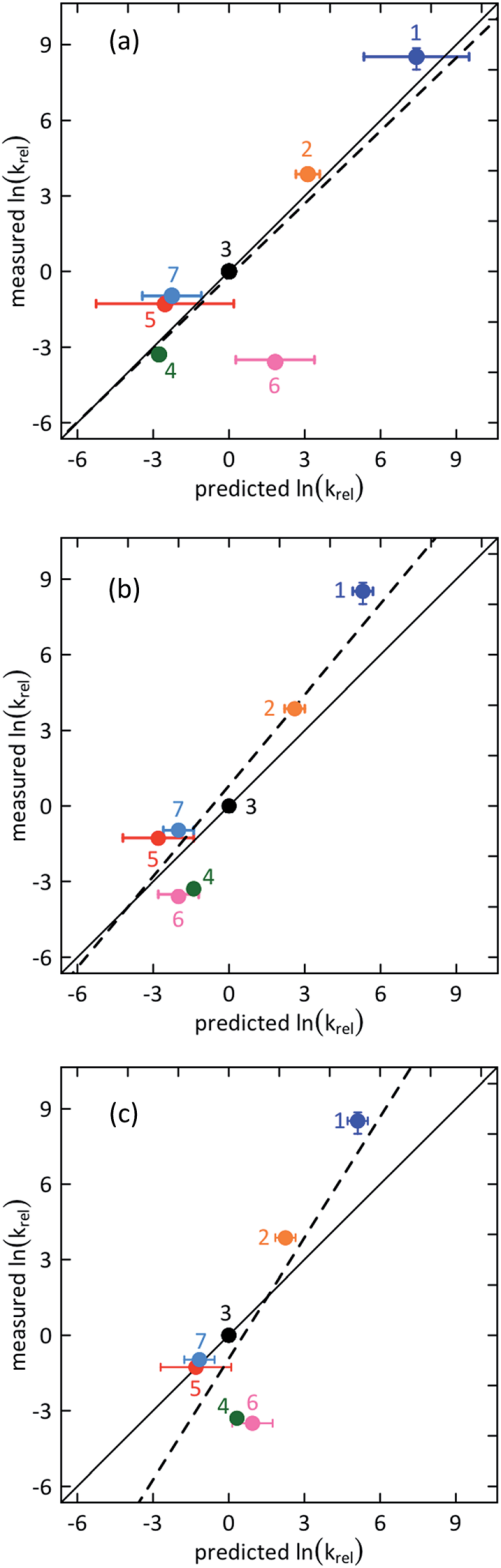
Correlation plots for measured against predicted values of the second order PCET rate constant ln(*k*_rel_), relative to that for phenol 3, for (a) reversible ETPT (*r*^2^ = 0.91) (b) irreversible ETPT (*r*^2^ = 0.86) and (c) CEP (*r*^2^ = 0.71) reaction mechanisms (*r*^2^ values relate to the solid line). The broken line shows a linear fit to the data points and the solid line is drawn along the diagonal representing an ideal correlation of slope = 1. The numbering of phenols follows Chart 1. The vertical error bars represent the 95% confidence interval (Table S1†). The horizontal error bars reflect the variation in literature values for E^0^_red_ and p*K*_a_ (Table S3†).

The rate of phenol oxidation depends upon the pH of the medium due to the presence of a pH dependent, very reactive phenolate ion (PhO^−^) species. However, in the low pH range of 0–2 (pH = 0 in the case of 3F and 2,6-F) the rate of phenol oxidation is entirely due to the protonated (PhOH) species, as seen by a pH-independent reaction rate. Phenols 8–12 did not show pH independent rates even at pH = 0 and were excluded from the correlation in Fig. 2. The PCET mechanistic studies were performed in neat water as a solvent to ensure that water was the only proton acceptor in the system. The pH was adjusted using HCl or H_2_SO_4_ and NaOH. At this low pH the buffer capacity of water is sufficient for convenient pH stability.

The mechanism can be deduced by analysis and comparison of the observed variation in the PCET rate constant for the series of phenols with that predicted for the respective mechanism of ETPT and CEP (Fig. 2). As is customary for ET and PCET reactions with an homologous series of reactants, we assume that the stability constant of the precursor and successor complexes is invariant,^34,40^ and that the variation in reorganization energy (*λ*) between the phenols is small; the aqueous *λ* value of PhOH–PhOH^+^˙ for 1 and 3 has indeed been reported to be equal within 5%.^41^ In the following paragraphs we derive the predicted variations before we comment on the results of Fig. 2.

At pH = 0–2, phenol deprotonation will occur by water (H_2_O) and not by OH^−^ (eqn (1)). Because of the large Δp*K*_a_ between the conjugate acid H_3_O^+^ (p*K*_a_ = 0) and phenol (p*K*_a_ = 10), PTET where water is a proton acceptor is too slow to be consistent with the observed rate constant (*k*_PT_ = 10^11−p*K*_a_^ = 10 s^−1^).^42–44^ Moreover, the phenols with lower p*K*_a_ values give slower *k*_obs_, in contradiction with a PTET mechanism. Therefore, the PTET mechanism can be ruled out of this discussion.1
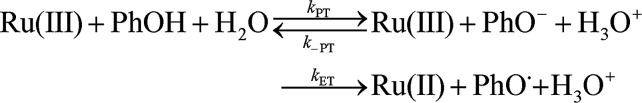


In the case of ETPT (eqn (2)), the rate constant is not pH dependent, as p*K*_a_ < −1 for all the PhOH^+^˙ studied and deprotonation to H_2_O dominates, but rather it depends upon the substituents on the phenol. This mechanism can be studied in terms of two separate stepwise mechanistic regimes, namely, irreversible ETPT and reversible ETPT.2
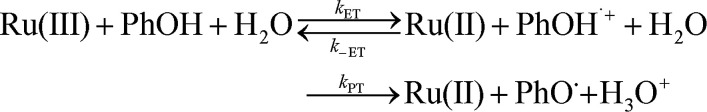


### Reversible ETPT

In the case where reversible ET is followed by PT (*k*_−ET_ ≫ *k*_PT_), the overall rate constants can be derived from pre-equilibrium kinetics, which yields; *k*_obs_ = *k*_ET_/*k*_−ET_·*k*_PT_. Here *k*_ET_/*k*_−ET_ is decreased by a factor of 10 for each 59 meV increase in Δ*G*^0^_ET_. Deprotonation of an Eigen acid in water follows *k*_PT_ ≈ 10^11−p*K*_a_^ s^−1^.^8^ This results in the following rate constant expression for reversible ETPT (p*K*_a_ refers here to the PhOH^+^˙ species):3a
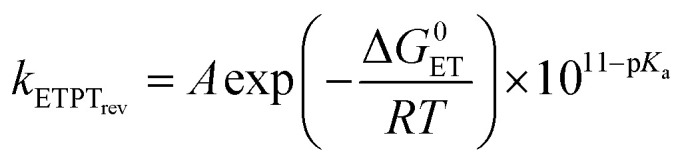
3b
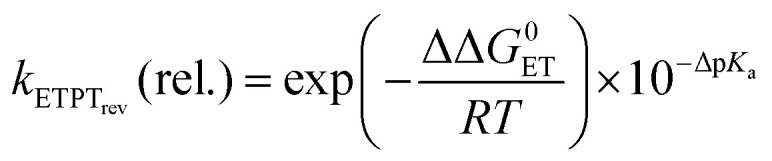
where *k*_ETPT_rev__(rel.) = *k*_ETPT_rev__/*k*_ETPT_rev__ (phenol 3), ΔΔ*G* and Δp*K*_a_ is the value relative to phenol 3, *A* is a pre-exponential factor and the p*K*_a_ values can be obtained from the literature (Table S2†). Eqn (4) was employed to obtain the driving force for the ET step, Δ*G*^0^_ET_ with an assumption that the coulombic interaction between the involved species is negligible.4
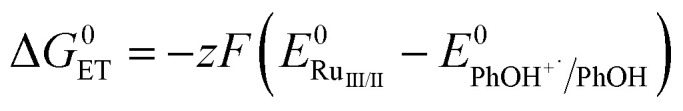
here, *z* is the number of electrons transferred, *E*^0^_RuIII/II_ = 1.26 V *vs.* NHE^45^ and 
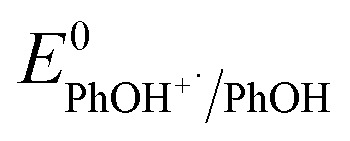
 is the pH-independent potential of the substituted phenol obtained from the literature or experimentally determined in this work (Table S3†).

### Irreversible ETPT

Following eqn (2), the oxidation of phenol leads to a significant drop in its p*K*_a_ to around −2. As a result the PhOH^+^˙ species is rapidly deprotonated which could make electron transfer the rate limiting step (*k*_−ET_ ≪ *k*_PT_ in addition to *k*_ET_ ≪ *k*_PT_). This results in *k*_obs_ = *k*_ET_. The rate of irreversible ETPT can thus be determined by a standard Marcus-type rate expression (eqn. (5)). The value relative to that for phenol 3 was calculated using eqn (5b);^46^ this assumes that |Δ*G*^0^_CEP_| ≪ *λ*_CEP_, which is reasonable as Δ*G*^0^_ET_ varies from −0.34 to 0.00 eV in the series of phenols 1–7.5a
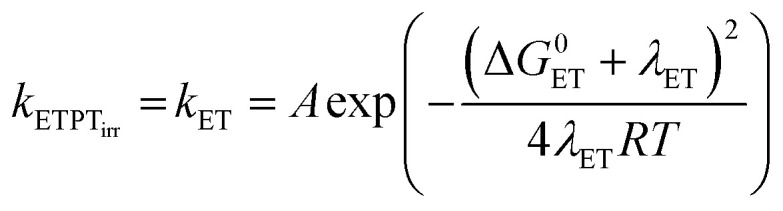
5b
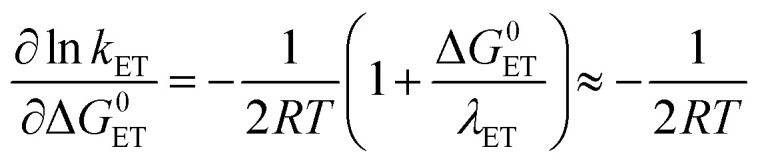
Here *A* is the pre-exponential factor and *λ*_ET_ is the reorganizational energy for the electron transfer step, which is assumed to be constant for the series of phenols. Eqn (5b) gives the predicted dependence of ln *k*_ETPT_irr__ (=ln *k*_ET_) on the driving force for the series of phenols.

### Concerted mechanism (CEP)

In this mechanism, electron and proton transfer occurs in a single kinetic step (eqn (6)).6
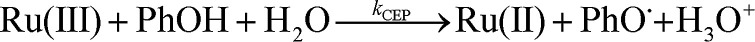


**Scheme 1:**
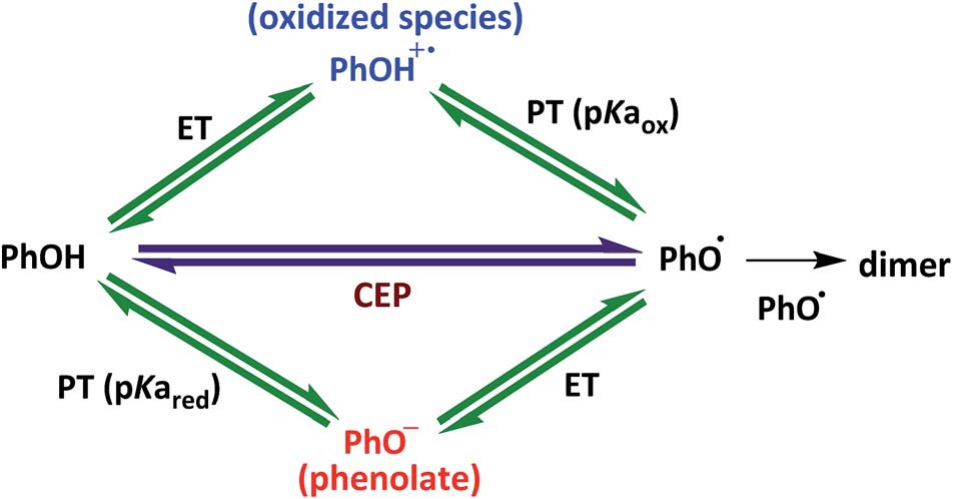
The mechanism of phenol oxidation *via* ETPT, PTET and CEP pathways.

The driving force for CEP with water as an acceptor is equal to the sum of the driving forces for oxidation to PhOH^+^˙ (eqn (4)) and its subsequent deprotonation (upper path in Scheme 1):7
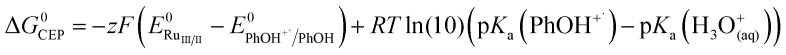
with p*K*_a_(H_3_O_(aq)_^+^) = 0. Analogous to ETPT_irr_, the rate constant for CEP can be determined by using a Marcus-type rate expression (eqn (8)), assuming |Δ*G*^0^_CEP_| ≪ *λ*_CEP_ in eqn (8b).^46,47^ We note that a value of *λ*_CEP_ = 0.45 eV for the reaction between [Ru(bpy)_3_]^3+^ and 3 in water has been suggested;^8^ however, the use of that value to predict relative values of *k*_CEP_ resulted in a poor correlation with experimental data, with a curvature that clearly suggests that the value of *λ*_CEP_ should be significantly larger than 0.45 eV, see Fig. S4.†8a
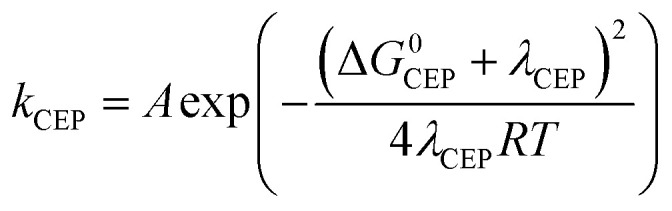
8b
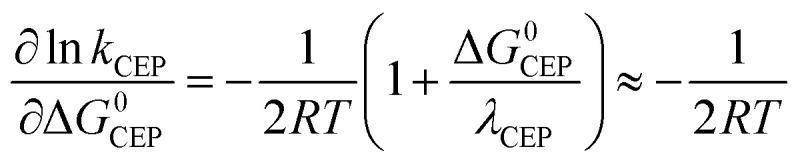


Fig. 2 shows the correlation of experimental *vs.* calculated rate constants relative to the value for the case of unsubstituted PhOH; hence PhOH is distinctly represented in the centre of all plots. The relative rate constants for the three mechanisms were calculated according to eqn (3a), (3b), (5a), (5b), (8a) and (8b), using reduction potentials and p*K*_a_ values of phenol derivatives (Table S3†). The horizontal error bars on the data points are associated with the uncertainty in the literature values for *E*^0^ and p*K*_a_ of the studied phenols.^48–50^ The operative mechanism under the present conditions should be revealed by a good correlation between experimental and predicted values. We point out that the plots for irreversible ET and CEP (Fig. 2b and c, respectively) are identical to their respective Marcus plots of ln *k vs.* Δ*G*^0^, with a linear transformation of the *x*-axis according to eqn (5b) and (8b), respectively (1 ln-unit = 50 meV driving force), and the solid line shows the slope of 1/(50 meV) predicted by eqn (5b) and (8b).

A simple inspection of the three correlation plots (Fig. 2) indicates that the best agreement between experimental and predicted relative rate constants is in the case of ETPT, while for CEP the correlation is comparatively poor. More precisely, a linear regression analysis reveals that in the case of reversible ETPT (Fig. 2a) the data points obey linearity with respect to the unsubstituted phenol (point 3) with a slope of ≈1. This is illustrated by the nearly perfect overlap between the linear fit (dashed line) and ideal correlation (solid line). The irreversible ETPT mechanism (Fig. 2b) shows the second best correlation with a slope of 1.2 and some deviation of the data points from the reference diagonal line. On the other hand, the slope of 1.6 for the CEP mechanism (Fig. 2c) shows a strong deviation from the typical Marcusian rate dependence on the CEP driving force. Phenols with both the highest and lowest observed rate constants deviate substantially from the predicted values in this case.

From the above comparison of the correlations in Fig. 2, we draw the conclusion that the PCET reaction for this series of phenols with [Ru(bpy)_3_]^3+^ in water follows a step-wise, ETPT mechanism. Regarding which of the two kinetic limits is most likely, we note that reversible ETPT (Fig. 2a) shows a slightly better agreement with predictions than the irreversible ETPT (Fig. 2b). On the other hand, this would require that the reverse ET in the solvent cage is faster than deprotonation of the PhOH^+^˙ intermediate, which occurs with *τ* = 0.1–1 ps (*k*_PT_ ≈ 10^11−p*K*_a_^ s^−1^).^8^ While this cannot be excluded, it is questionable that this would hold for the entire series of phenols with different potentials and p*K*_a_ values. It is of course possible that the mechanism gradually changes between ETPT_rev_ and ETPT_irrev_ within the series. In the absence of clearer proof, we leave it as an open question whether the step-wise ETPT mechanism follows an irreversible or a reversible pathway.

## Conclusions

A central conclusion that can be drawn at this juncture is that the oxidation of the series of phenols by [Ru(bpy)_3_]^3+^ shows that a stepwise ETPT mechanism is most likely to occur at low pH with water as a proton acceptor. This conclusion is based on rate correlations of phenols yielding rate constants that vary from 1 × 10^5^ to 2 × 10^9^ M^−1^ s^−1^. The results are in line with our previous assignment for intramolecular PCET in a Ru–Tyrosine complex, where at low pH a stepwise ETPT mechanism was proposed when water is the proton acceptor.^10^ In contrast the Marcus-type analysis suggests that the operating mechanism under the conditions investigated is not a concerted reaction (CEP), as was suggested before, and as a consequence the reorganization energy value of 0.45 eV reported is not correct.^8^ This impacts our understanding of the competition between concerted and step-wise PCET mechanisms of tyrosine and other phenols in water. The mechanistic investigation using a Marcus-type relationship offers a successful tool to discern among these PCET mechanisms. This study provides insight from a model system into the mechanisms of PCET for the working of various biological processes where phenoxyl radicals play a pivotal role.

## Supplementary Material

SC-007-C6SC00597G-s001
