# Supplementary material for: Marcus-type driving force correlations reveal the mechanism of proton-coupled electron transfer for phenols and [Ru(bpy)3]3+ in water at low pH
Source: Chem Sci. 2016 Apr 1;7(7):4607–12. doi: 10.1039/c6sc00597g (PMC6013771; doi:10.1039/c6sc00597g)
Supplement: SC-007-C6SC00597G-s001 [file SC-007-C6SC00597G-s001.pdf]

Electronic Supplementary Material (ESI) for Chemical Science.
This journal is © The Royal Society of Chemistry 2016

Supplementary Material for

Marcus-type Driving Force Correlations Reveal the Mechanism of Proton-Coupled Electron Transfer for Phenols and $[Ru(bpy)_3]^{3+}$ in Water at Low pH

Janne Soetbeer, Prateek Dongare★ and Leif Hammarström★

Department of Chemistry - Ångström Laboratory, Uppsala University, Box 523, SE-751 20, Uppsala, Sweden

leif.hammarström@kemi.uu.se; prateek.dongare@kemi.uu.se

Table of Contents

1. List of Figures
2. Experimental Procedures
   2.1. Sample Preparation
   2.2. Data Acquisition
3. Transient Absorption Traces at 410 and 450 nm.
4. Table of Rate Constants
5. Transient Absorption Spectra
6. Dependence of k_{obs} on Phenol Concentration
7. Mechanism of PCET
   7.1. Irreversible ETPT
   7.2. Irreversible ETPT
   7.3. CEP Mechanism
8. Electrochemical Data
   8.1. p*K*a values and reduction potentials E^0_{red} for the studied phenols
   8.2. Brown's σ^+ Constants vs E^0_{red} Correlations
9. References

1. List of Figures

Figure S1. Representative transient absorption kinetic traces and the corresponding fits for $[Ru^{II}(bpy)_3]$ and (a) 4-MeO, pH = 3 (b) 4-Me, pH, = 4 (c) PhOH, pH = 3 (d) 2-Cl, pH = 1 (e) 2-F, pH = 1 (f) 3-F, pH = 2 (g) 2,6-F, pH = 1 in presence of $[Co(NH_3)_5Cl]^{2+}$ as external oxidant. Data is collected by exciting at 460 nm. Blue curve is a fit to $[Ru(bpy)_3]^{2+}$ -

$[Ru(bpy)_3]^{3+}$ absorption bleach at 450 nm and red curve is a fit to 410 nm transient absorption, which is due to a combination of $[Ru(bpy)_3]^{3+}$ - $[Ru(bpy)_3]^{2+}$ bleach recovery and $PhO^{\cdot}$ induced absorption. [MeO] = 1.20, [4-Me] = 1.80, [PhOH] = 2.0, [2-Cl] = 2.0, [2-F] = 3.20, [3-F] = 3.20, [2,6-F] = 0.9 mM.

Figure S2. Transient absorption spectra of $[Ru(bpy)_3]^{2+}$ acquired by exciting at 532 nm using 10 ns laser pulse at 298 ± 2 K in presence of (a) phenol 3 in pH 2 water (b) phenol 7 in pH 1 water and $[Co(NH_3)_5Cl]^{3+}$ as an external electron acceptor.

Figure S3. Plots of (a) phenol 1, MeO (b) phenol 2, Me (c) phenol 3, PhOH (d) phenol 5, 2-F (e) phenol 6, 3-F; showing the dependence of k_{obs} on the concentration of phenol at pH = 2.

Figure S4. Correlation plots for measured vs. predicted values of the second order rate constants $ln(k_{rel})$, relative to that for **3**, as in Figure 2. This correlation assumes a CEP reaction with λ_{CEP} = 0.45 eV as suggested in ref. 8 of the main paper. The solid line is drawn along the diagonal, representing an ideal correlation.

Figure S5. A: CV scan (ν = 0.17 Vs^{-1}) for 0.37 mM phenol at pH 12 in 0.12 M $[(C_2H_5)_4NCl]$. **B**: *E*p as a function of ln(ν) for phenol (black), 3-F (orange), 2,6-F (red) and 3,5- F (brown) at pH 12.

Figure S6. A plot of E^0_{red} (pH 12) as a function of Brown's σ^+ constants for chlorosubstituted phenols as reported by Hoffmann *et al.*[S15] and experimentally determined E^0_{red} for fluorophenols. The dashed line corresponds to the linear correlation E^0_{red} = 0.81+0.26 reported by Hoffmann *et al.*[S15] and solid line shows the correlation obtained for the fluorosubstituted phenols. Fluoro and chloro phenols are represented by spheres and squares, respectively. 3 = H, 4 = 2-Cl, 5 = 2-F, 6 = 3-F, 7 = 2,6-F, 9 = 2,5-Cl, 10 = 3,5-F, 11 = 3-Cl, 12 = 2,6-Cl.

2. Experimental Procedures

2.1. Sample Preparation

The aqueous solutions were prepared using deionized water (17 MΩ cm). Isotopic effect measurements were conducted using deuterium oxide (Sigma Aldrich, 99.9%) as solvent. $[Ru(bpy)_3]Cl_2.6H_2O$ (Acros), phenols (Sigma Aldrich, ≥ 99%) and external quenchers $[Co(NH_3)_5Cl]^{2+}.Cl_2$ (Alfa Aeser) was used as received and Methylviologen dichloride hydrate (Aldrich, 98%) was recrystallized from ethanol. The pH was adjusted with NaOH(aq) and concentrated HCl or H_2SO_4 prior to and during the data acquisition using a Methrom pH-meter (Micro electrode). In a typical flash-photolysis experiment the sample contained 5-10 mM of $[Co(NH_3)_5Cl]^{3+}$, 25 μM of $[Ru(bpy)_3]^{2+}$ and 0.25-3.25 mM of a phenol. The samples were degassed with N_2 gas before and during the measurements. All the measurements involving $[Ru(bpy)_3]^{2+}$ and $[Co(NH_3)_5Cl]^{3+}$ were carried out in dark to ensure that the excitation of $[Ru(bpy)_3]^{2+}$ is exclusively achieved by laser flash and the effect of ambient light was therefore eliminated. Some of the measurement were extended up to 400 ms and the control experiments in the absence of phenol show that $[Ru(bpy)_3]^{3+}$ is stable on the long time scale at pH = 2, as also demonstrated by Glover *et al.*[S1]

2.2. Data Acquisition

Optical absorption spectra were measured on a Cary 5000 instrument from Varian before and after the experiments in order to verify that no sample degradation had occurred during irradiation. By optical absorption spectroscopy we also ensured that there are no significant absorbing impurities present in the samples.

For transient absorption measurements, optical excitation was afforded by an OPO (opotek) pumped by a frequency tripled Q-switched Nd:YAG laser (Quantel Brilliant B), delivering c.a. 7 ns pulses at 460 nm (c.a. 20 mJ/pulse). A pulsed Xenon lamp of an Applied Photophysics LKS60 setup provided analyzing light that was passed through 1×1 cm quartz sample cuvette in a right-angle configuration and through a monochromator before hitting the

P928 type photomultiplier. The PMT signal was converted and digitized using a HP Infinitum digital oscilloscope (2G samples/s). Transient absorption traces were generated from the raw data used the LKS60 software.

Electrochemical measurements were carried out using a PC-controlled three-electrode system connected to an Eco Chemie model Autolab in combination to a GPES software program. The electrode setup consisted of a glassy carbon working electrode, the reference electrode, an aqueous Ag/AgCl in saturated KCl, and a platinum counter electrode. Both reference and counter electrodes were separated from the sample solution by porous glass membranes. Before every measurement the working electrode was thoroughly polished with 0.3 μm and then 0.05 μm Buehler MicroPolish, sonicated and rinsed with methanol and deionized water to remove deposited products from previous measurements.

3. Transient absorption traces at 410 and 450 nm.

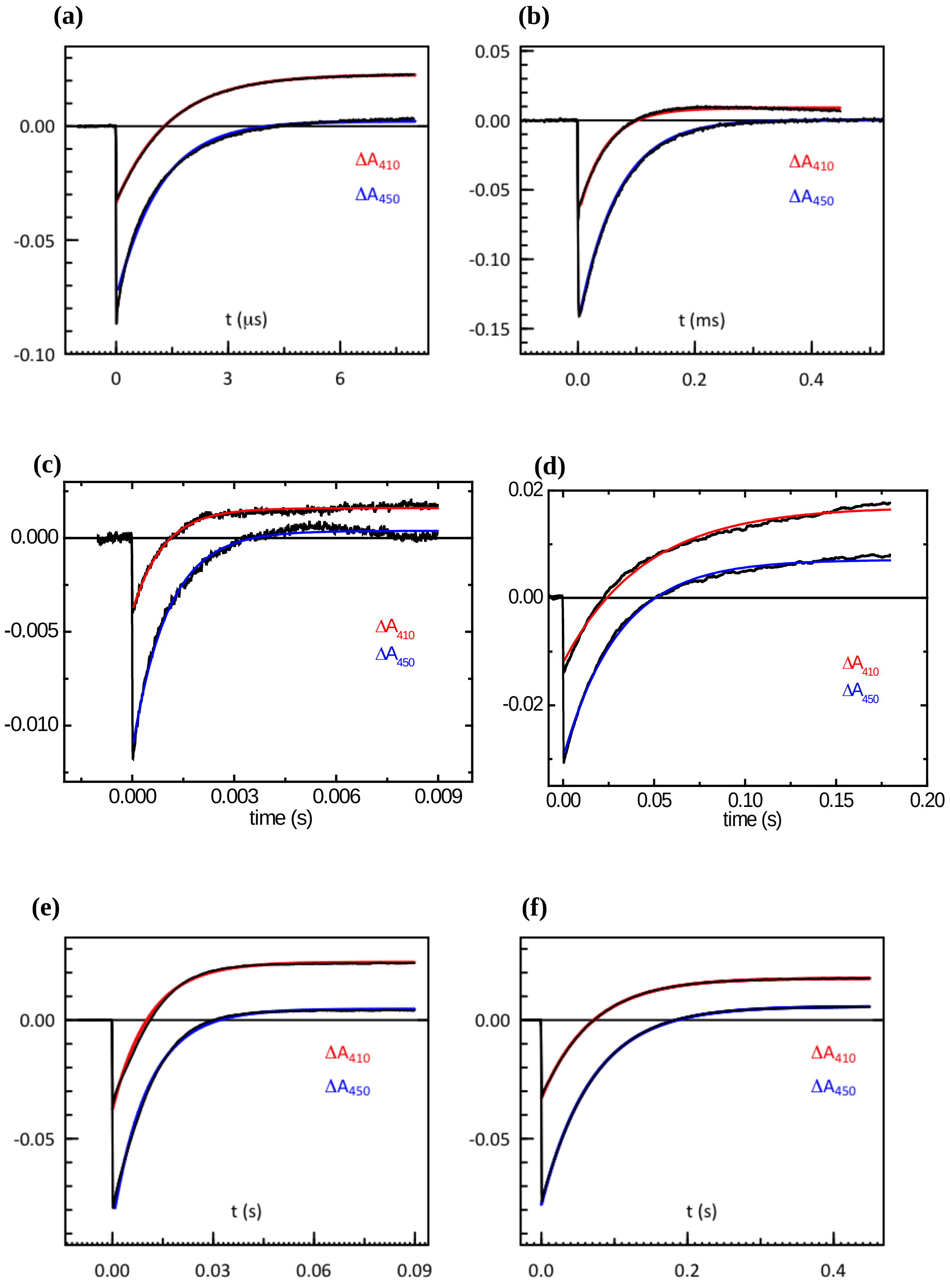

(g)

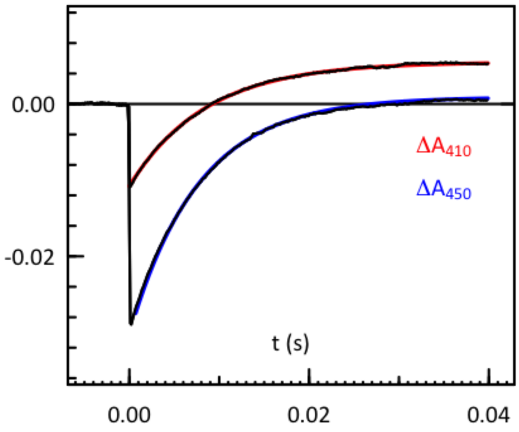

Figure S1. Representative transient absorption kinetic traces and the corresponding fits for $[Ru^{II}(bpy)_3]$ and (a) 4-MeO, pH = 3 (b) 4-Me, pH, = 4 (c) PhOH, pH = 3 (d) 2-Cl, pH = 1 (e) 2-F, pH = 1 (f) 3-F, pH = 2 (g) 2,6-F, pH = 1 in presence of $[Co(NH_3)_5Cl]^{2+}$ as external oxidant and the Y-axes in the plots read ΔOD. Data is collected by exciting at 460 nm. Blue curve is a fit to $[Ru^{II}(bpy)_3]$ - $[Ru^{III}(bpy)_3]$ absorption bleach at 450 nm and red curve is a fit to 410 nm transient absorption, which is due to a combination of $[Ru(bpy)_3]^{3+}$ - $[Ru(bpy)_3]^{2+}$ bleach recovery and $PhO^{\bullet}$ induced absorption. [MeO] = 1.20, [4-Me] = 1.80, [PhOH] = 2.0, [2-Cl] = 2.0, [2-F] = 3.20, [3-F] = 3.20, [2,6-F] = 0.9 mM.

4. Table of rate constants

Table S1. List of k_{PCET} reaction rates with 95% confidence interval. Please see chart 1 to follow the numbering of phenol type.

Phenol type	pH	k_{PCET} (M^{-1} s^{-1})	
		410 nm ***(±95% conf.int.)***	450 nm ***(±95% conf.int.)***
1	2	$1.7(\pm 0.6) \times 10^9$	$1.9(\pm 0.7) \times 10^9$
	3	$1.23(\pm 0.03) \times 10^9$	$1.49(\pm 0.11) \times 10^9$
	4	$1.27(\pm 0.03) \times 10^9$	$1.44(\pm 0.02) \times 10^9$

Phenol type	pH	k_{PCET} (M^{-1} s^{-1})	
		410 nm ***(±95% conf.int.)***	450 nm ***(±95% conf.int.)***
2	2	$2.88(\pm 0.11) \times 10^7$	$1.81(\pm 0.20) \times 10^7$
	3	$2.25(\pm 0.16) \times 10^7$	$1.79(\pm 0.14) \times 10^7$
	4	$2.53(\pm 0.03) \times 10^7$	$1.73(\pm 0.23) \times 10^7$

Phenol type	pH	k_{PCET} (M^{-1} s^{-1})	
		410 nm ***(±95% conf.int.)***	450 nm ***(±95% conf.int.)***
3	2	$3.6(\pm 0.6) \times 10^5$	$3.8(\pm 0.6) \times 10^5$
	3	2.5×10^5	3.0×10^5
	4	$3.7(\pm 0.6) \times 10^5$	6.3×10^5

Phenol type	pH	k_{PCET} (M^{-1} s^{-1})	
		410 nm ***(±95% conf.int.)***	450 nm ***(±95% conf.int.)***
4	0	$1.36(\pm 0.07) \times 10^4$	$1.42(\pm 0.08) \times 10^4$
	1	$2.01(\pm 0.19) \times 10^4$	$2.25(\pm 0.10) \times 10^4$

Phenol type	pH	k_{PCET} (M^{-1} s^{-1})	
		410 nm ***(±95% conf.int.)***	450 nm ***(±95% conf.int.)***
5	0	$1.05(\pm 0.04) \times 10^5$	$1.05(\pm 0.03) \times 10^5$
	2	$9.2(\pm 0.4) \times 10^4$	$9.5(\pm 0.4) \times 10^4$

Phenol type	pH	k_{PCET} (M^{-1} s^{-1})	
		410 nm ***(±95% conf.int.)***	450 nm ***(±95% conf.int.)***
6	0	$1.29(\pm 0.04) \times 10^4$	$1.05(\pm 0.04) \times 10^4$
	1	$1.62(\pm 0.19) \times 10^4$	$1.57(\pm 0.11) \times 10^4$
	2	$1.86(\pm 0.017) \times 10^4$	$1.85(\pm 0.016) \times 10^4$

Phenol type	pH	k_{PCET} (M^{-1} s^{-1})

		410 nm *(±95% conf.int.)*	450 nm *(±95% conf.int.)*
7	0	$8.8(\pm1.4)\times10^4$	$1.0(\pm2.3)\times10^5$
	1	$1.32(\pm0.23)\times10^5$	$1.45(\pm0.04)\times10^5$

There is no trend in the data that suggests substantial involvement of the PhO^- species in the reaction at pH = 0 (pH = 2 for **1-3**). If PhO^- would be the only reacting species the observed rate constant would decrease by one order of magnitude from pH=1 to pH = 0. Instead, we see at most a ±20% variation (increase as well as decrease) over two pH units for **1-3** and **5**. For **4**, **6** and **7** we see no more than a 40% decrease in rate constant at 450 nm from pH = 1 to pH = 0. Thus, even if only PhO^- were responsible for the reaction at pH = 1, its contribution at pH = 0 would be less than 20%. A ±20% variation equals ca. ±0.2 ln-units, meaning that this uncertainty is not greater than the size of the data points in Figure 2.

5. Transient absorption spectra

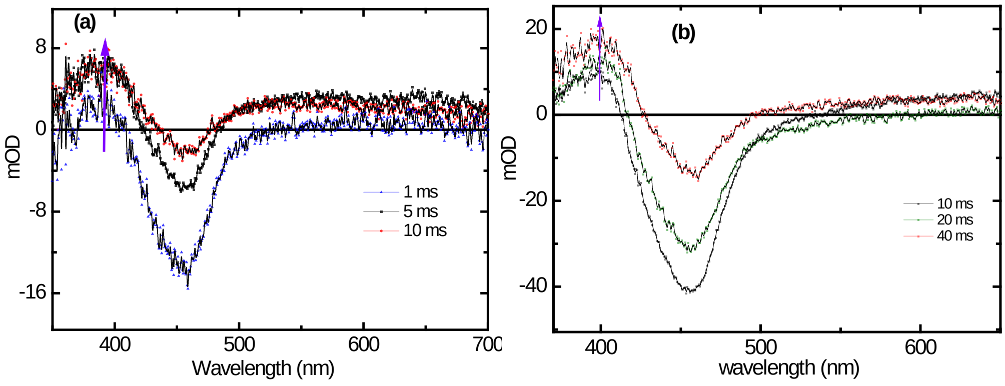

Figure S2. Transient absorption spectra of $[Ru(bpy)_3]^{2+}$ acquired by exciting at 532 nm using 10 ns laser pulse at 298 ±2 K in presence of (a) phenol **3** in pH 2 water (b) phenol **7** in pH 1 water and $[Co(NH_3)_5Cl]^{3+}$ as an external electron acceptor.

The unsubstituted phenoxyl radical (deprotonated radical of **3**) and its analog tyrosine radical show an absorption band around 410 nm and a weaker, relatively broad band in the region of *ca.* 450-700 nm.[S1-S4] Figure S2 shows examples of transient spectra for the PCET reaction of

the substituted phenols. All phenols showed the same basic features: the Ru^{II} ground state bleach around 450 nm recovered simultaneously with an increase in the phenoxyl radical bands around 400 nm and further to the red. For technical reasons, we could not record reliable transient *spectra* on a time scale longer than *ca.* 40 ms, but from the transient *traces* at 410 nm we see we see that the radical is long-lived compared to the PCET reaction. This is the reason that a weak positive absorption is seen from several of the phenoxyl radical even at 450 nm when all Ru^{II} has recovered (Figure S1). The data also shows that its subsequent and slow decay at low pH, presumably by dimerization, does not affect the PCET kinetics. The long lived nature of phenoxyl radicals at low pH (pH of around 2) has been debated earlier in the literature,[S5,S6] but this is not the scope of the present paper.

6. Dependence of k_{obs} on phenol concentration

The plots below shows the linear dependence of pseudo-first order rate constant obtained by single exponential fit of 450 nm kinetic trace on the phenol concentration and the rate constants are provided in Table S2.

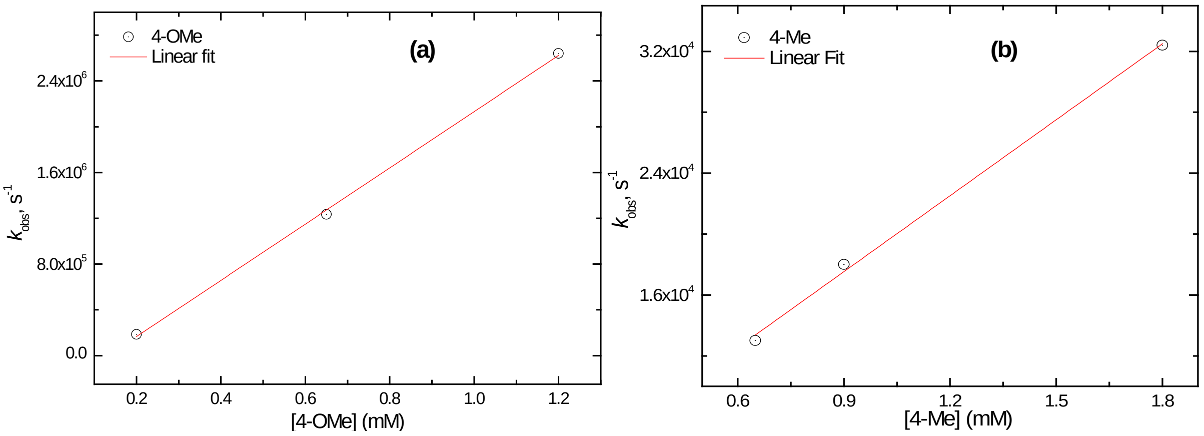

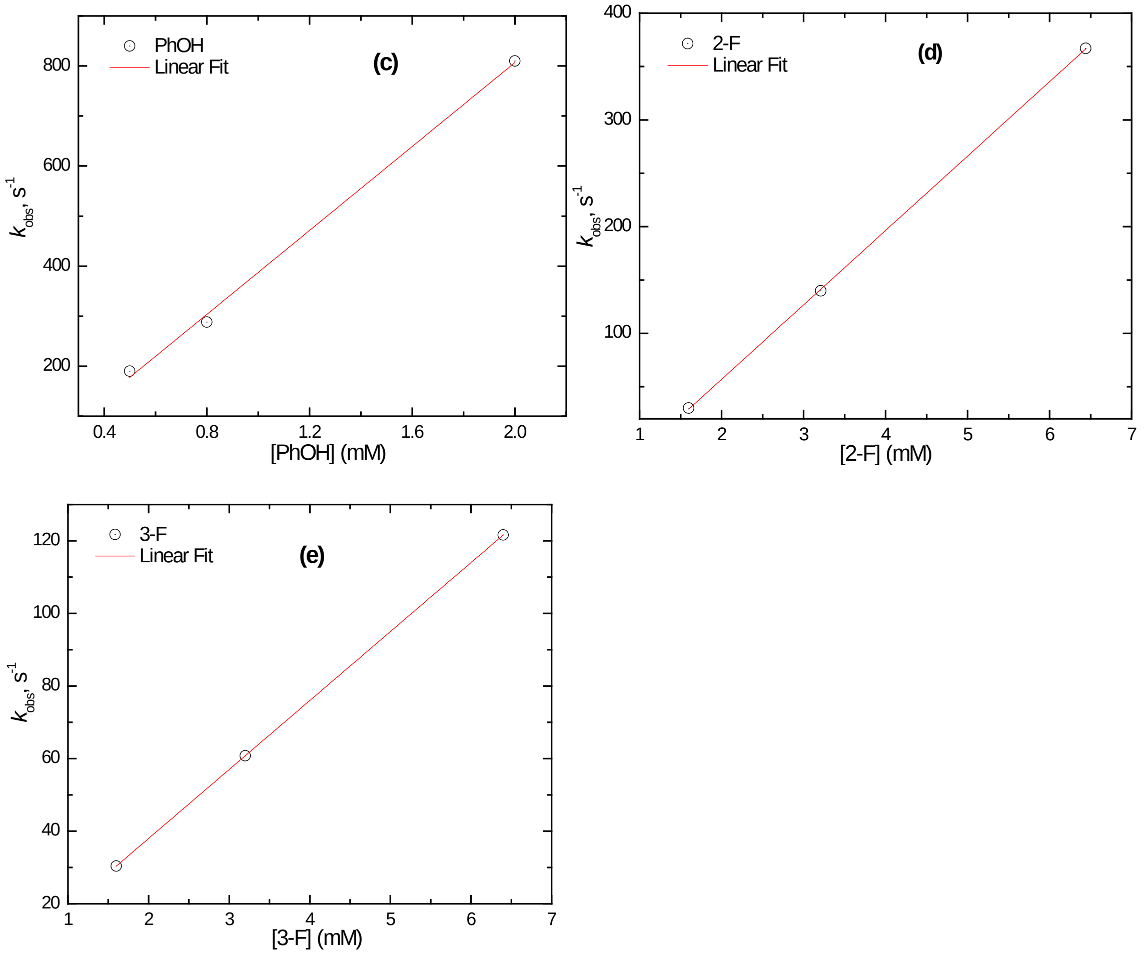

Figure S3. Plots of (a) phenol 1, MeO (b) phenol 2, Me (c) phenol 3, PhOH (d) phenol 5, 2-F (e) phenol 6, 3-F; showing the dependence of k_{obs} on the concentration of phenol at pH = 2.

Table S2. The Table of second order rate constants extracted from the linear fits of figure S3.

Phenol Derivative	k_{obs} (M^{-1} s^{-1})
**1**	2.50 (±0.6) × 10^{9}
**2**	1.67 (±0.14) × 10^{7}
**3**	4.19 (±0.4) × 10^{5}
**5**	6.80 (±0.04) × 10^{4}
**6**	1.90 (±0.05) × 10^{4}

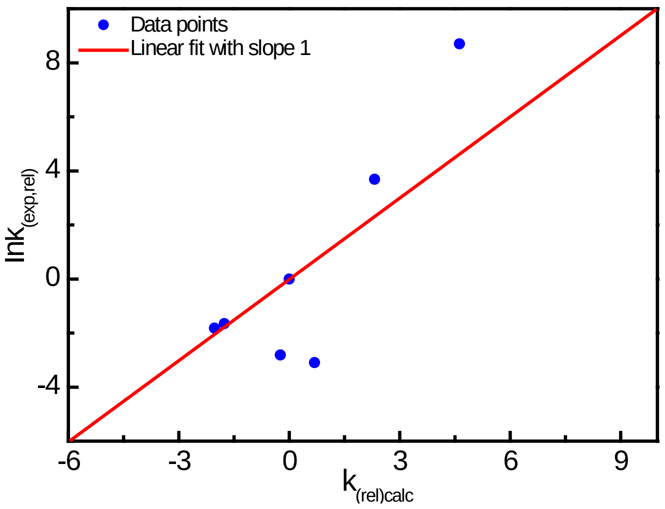

Figure S4. Correlation plots for measured vs. predicted values of the second order rate constants $\ln(k_{rel})$, relative to that for **3**, as in Figure 2. This correlation assumes a CEP reaction with λ_{CEP} = 0.45 eV as suggested in ref. 8 of the main paper. The solid line is drawn along the diagonal, representing an ideal correlation.

Relative rate constants were calculated using eq. 8a of the main paper, and a value of ΔG^0 = 0.080 eV and λ_{CEP} = 0.45 eV for the reaction of **3**, as given in ref. 8. For the other phenols ΔG^0 relative to that for **3** was calculated from the relative values of $E^0_{PhOH^{+\bullet}/PhOH}$ and p*K*a($PhOH^{+\bullet}$), according to Table S3.

The correlation with experimental data is poor, as shown by the deviation from the ideal diagonal (solid line). The data also show an upward curvature, meaning that the predicted rate constants increase more slowly than the experimental ones, as the driving force increases. This is expected, from eq. 8b of the main paper, when the reorganization energy is underestimated. The data support our assumption that $-\Delta G^0 << \lambda$ for these weakly exergonic reactions ($\Delta G^0 > -0.25$).

7. Mechanism of PCET

The mechanism of PCET in this system follows the events portrayed in scheme S1. The kinetics was studies in the range of pH 0-4 in aqueous solvent to ensure a pH-independent rate constant with influence of the reactive phenolate species. For phenols **8** and above a pH-

independent rate constant was not reached even at pH = 0 and these were therefore excluded from the correlation.

Owing to the chosen experimental conditions i.e. [PhOH]>> $[Ru(bpy)_3]^{3+}$, all the rate constants, k_{obs}, described in this section are pseudo first order rate constant k_{obs} in s^{-1}, the PCET reaction is bimolecular which can be calculated by eq. S1.

$$k_{\mathrm{PCET}} = \frac{k_{\mathrm{obs}}}{[\mathrm{PhOH}]} \qquad [S1]$$

As PTET can be excluded at these pH values, the PCET mechanism can be classified into either concerted (CEP) or stepwise (ETPT) mechanism. Furthermore, depending upon the initial step of the electron transfer, ETPT can be divided into reversible or irreversible. Therefore the rates are expected to be different for irreversible and reversible ETPT mechanism.

7.1. Irreversible ETPT mechanism

An irreversible ETPT mechanism proceeds as per eq. S2

$$\mathrm{Ru(III)} + \mathrm{PhOH} \xrightarrow{k_{\mathrm{ET}}} \mathrm{Ru(II)} + \mathrm{PhOH}^{\bullet+} \xrightarrow{k_{\mathrm{PT}}} \mathrm{Ru(II)} + \mathrm{PhO}^{\bullet} + \mathrm{H}^{+} \qquad [S2]$$

With p*K*a values of around -2 for the protonated phenoxyl radical, $PhOH^{+\cdot}$ species will be quickly deprotonated. In the case of a much slower reverse electron transfer to phenol in comparison to deprotonation (k_{PT}>>k_{-ET}), the overall observed reaction rate is given by k_{obs} = $k_{ET,}$ hence the electron transfer is the rate limiting step. The driving force for the electron transfer can be calculated by using eq. S3.

$$\Delta G^0_{\mathrm{ET}} = -zF\left(E^0_{\mathrm{Ru_{III/II}}} - E^0_{\mathrm{PhOH^{+\bullet}/PhOH}}\right) \qquad [S3]$$

where, z is the number of electron transferred in the reaction, F is the Faraday constant, $E^0_{\mathrm{Ru_{III/II}}}$ corresponds to the pH independent reduction potential of $[\mathrm{Ru(bpy)_3}]^{3+/2+}$, which is taken as 1.26 V vs. NHE in water,[S7] while $E^0_{\mathrm{PhOH^{+\bullet}/PhOH}}$ refers to the reduction potential of $\mathrm{PhOH^{+\bullet}/PhOH}$, which can be calculated by using eq. S4.[S8]

$$\mathrm{E^0_{PhOH^{+\bullet}/PhOH} = E^0_{PhO^\bullet/PhO^-} + \frac{RT}{zF} \cdot \ln(10) \cdot [p\mathit{K}_a(PhOH) - p\mathit{K}_a(PhOH^{+\bullet})]} \qquad [S4]$$

The values for p*K*a($\mathrm{PhOH^{+\bullet}}$) were obtained from the literature (Table S3) whereas the value for phenol **7** (2,6-F) was crudely estimated by a lower limit of -2 as per the following argument. The rate of deprotonation of an Eigen acid in H_2O has a rate constant of $\approx 1\times10^{13}$ s^{-1} at a p*K*a of -2,[S9] as this is close to the limit for a barrier-less reaction this should be close to an upper limit of k_{PT}, implying a lower effective p*K*a limit of -2. As $k_{obs} = k_{ET}$, an irreversible ETPT reaction rate should only vary with the reduction potential of the phenol. The rate of irreversible ETPT can be determined by using a standard Marcus-type rate expression (eq. S5a).[S10]

$$k_{ETPT_{irr}} = k_{ET} = A \cdot \exp\left(-\frac{\left(\Delta G^0_{ET} + \lambda_{ET}\right)^2}{4\lambda_{ET}RT}\right) \qquad [S5a]$$

from which the dependence of k_{ET} on ΔG^0_{ET} can be derived:

$$\frac{\partial \ln k_{ET}}{\partial \Delta G^0{}_{ET}} = -\frac{1}{2RT}\left(1 + \frac{\Delta G^0_{ET}}{\lambda_{ET}}\right) \approx -\frac{1}{2RT} \qquad [S5b]$$

7.2.Reversible ETPT mechanism

In the case where reversible ET is followed by PT ($k_{-ET} >> k_{PT}$), the overall rate can be derived from pre-equilibrium kinetics, which yields; $k_{\mathrm{obs}} = k_{\mathrm{ET}}/k_{-\mathrm{ET}} \cdot k_{\mathrm{PT}}$. Here $k_{\mathrm{ET}}/k_{-\mathrm{ET}}$ is decreased by a factor of 10 for each 59 meV increase in ΔG^0_{ET}. Deprotonation of an Eigen

acid in water follows $k_{PT} = 10^{11-pKa}$ s^{-1}. This results in the following rate constant expression for reversible ETPT (p*K*a refers here to the $PhOH^{+\bullet}$ species):

$$k_{ETPT_{rev}} = A \cdot exp\left(-\frac{\Delta G^0_{ET}}{RT}\right) \cdot 10^{11-pKa} \qquad [S6]$$

7.3.CEP mechanism

The driving force for CEP with water as proton acceptor is affected by both the reduction potential and p*K*a of the phenol species. It can be calculated as the sum of the driving forces for the two steps of oxidation to $PhOH^{+\bullet}$ (eq. S3) and subsequent deprotonation to water ($pK_a(H_3O^+) = 0$) of that species.

$$\Delta G^0_{CEP} = -zF\left(E^0_{Ru_{III/II}} - E^0_{PhOH^{\bullet+}/PhOH}\right) + RT\ln(10)\,pK_a(PhOH^{\bullet+}) \qquad [S7]$$

Employing Marcus-type rate expression similar to $ETPT_{irr}$, CEP rate constant can be deduced (eq. S8).[S10, S11]

$$k_{CEP} = A \cdot \exp\left(-\frac{\left(\Delta G^0_{CEP} + \lambda_{CEP}\right)^2}{4\lambda_{CEP}RT}\right) \qquad [S8a]$$

from which the dependence of k_{CEP} on ΔG^0_{CEP} can be derived:

$$\frac{\partial lnk_{CEP}}{\partial \Delta G^0{}_{CEP}} = -\frac{1}{2RT}\left(1 + \frac{\Delta G^0_{CEP}}{\lambda_{CEP}}\right) \approx -\frac{1}{2RT} \qquad [S8b]$$

8. Electrochemical data

A Table listing p*K*a values and reduction potentials E^0_{red} for the studied phenols as well as experimentally determined $E^0_{PhO^\bullet/PhO^-}$ are presented in the Table S3. A representative cyclic voltammogram (CV) (ν = 0.17 Vs^{-1}) for phenol at pH 12 which illustrates the irreversible

nature of the phenol oxidation is presented in fig S5a. Following literature procedures, E^0 for this irreversible reaction was determined from the scan rate dependence of the peak potential, according to eq. 10 (T = 296 K).[S12]

$$E_P = E^0_{red} + 0.902 \cdot \frac{RT}{F} - \frac{RT}{F} \cdot ln\left(\frac{2RTk_{dim}[PhOH]}{3F\nu}\right) \qquad [S9]$$

The phenoxyl radical dimerization constant $k_{\mathrm{dim}} = (2.6\pm0.3)\times10^9\ \mathrm{M^{-1}s^{-1}}$ [S5] and was assumed constant within the series, as was done before.[S13]

Fig S5b shows the consistency of the experimental data as plotting the one-electron potential *E*p against ln(ν) results in a linear relationship. However, the slope for 2-F deviates from that predicted by eq. 10 (8 mV/ln ν, or 20 mV/log ν). Therefore we also made an independent estimate of E^0 for 2-F by the correlation in Fig S6. The two methods result in the span of predicted rate constant for 2-F.

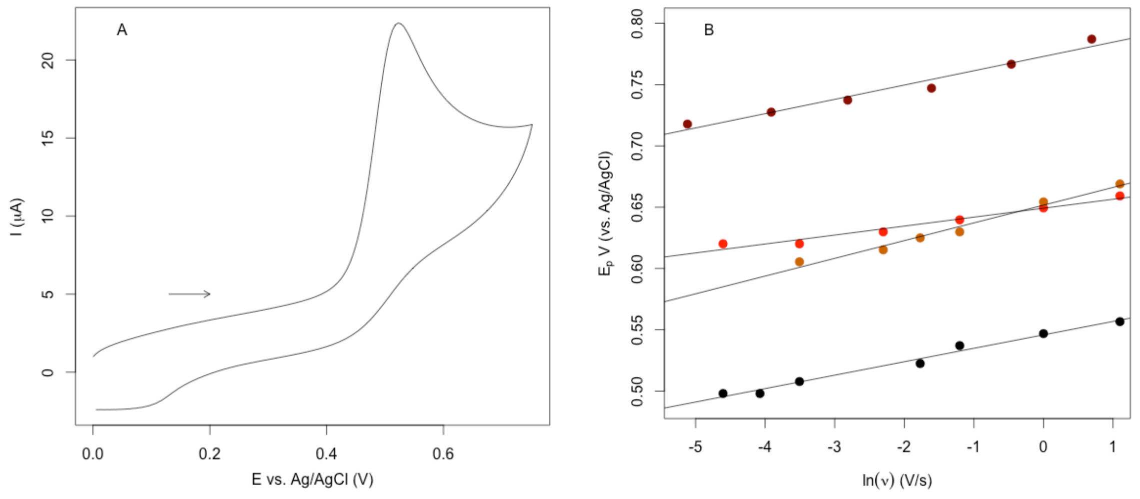

Figure S5. A: CV scan (ν = 0.17 Vs^{-1}) for 0.37 mM phenol at pH 12 in 0.12 M $[(C_2H_5)_4NCl]$. **B**: *E*p as a function of ln(ν) for phenol (black), 3-F (orange), 2,6-F (red) and 3,5- F (brown) at pH 12.

8.1. p*K*a values and reduction potentials E^0_{red} for the studied phenols

Table S3. p*K*a values and reduction potentials E^0_{red} for the studied phenols. Due to inconsistencies in the present literature values for phenol, 4-methyl and 4-methoxyphenol, two sets of p*K*a data were used in Fig 1 and are represented by horizontal uncertainty bars for the predicted ln(k_{PCET}) values.[S13-S15] Horizontal uncertainty bars for phenols 5-7 arise due to the 95% confidence interval associated with the experimental reduction potentials.

Phenol number as in Chart 1	Phenol type	p*K*a[a] (PhOH)	p*K*a[b] ($PhOH^{+\bullet}$)	$E^0_{PhO^\bullet/PhO^-}$ V (pH 11-12) *vs.* NHE[c]	$E^0_{PhO^\bullet/PhO^-}$ V (pH 11-12) *vs.* NHE[d]	$E^0_{PhO^\bullet/PhO^-}$, V (pH 12) *vs.* NHE[e]	$E^0_{PhOH^{+\bullet}/PhOH}$ V
1	4-MeO	10.20 (10.1)	-1.41	0.54 ± 0.02	0.58		1.24±0.04
2	4-Me	10.28 (10.3)	-1.60	0.68 ± 0.02	0.71		1.40±0.03
3	PhOH	9.98 (10.0)	-2.00	0.79 ± 0.01	0.86	0.85	1.54±0.04
4	2-Cl	8.52	-1.27		0.93		1.50
5	2-F	8.73	-1.69			0.99±0.07	1.60
6	3-F	7.51	-1.95			0.95±0.04	1.50
7	2,6-F	9.29	-2.00[f]			0.95±0.03	1.61

[(a)]Values are from Stradins, J.; Hasanli, B. *J. Electroanal. Chem.* **1993**, *353*, 57. Values in parentheses and [(b)] are from Dixon, W. T.; Murphy, D. *J. Chem. Soc., Faraday Trans. 2*, **1976**, *72*, 1221 [(c)] Taken from Lind, J.; Shen, X.; Eriksen, T. E.; Merenyi, G. *J. Am. Chem. Soc.* **1990**, *112*, 479 [(d)] Li, C.; M. Z. *J. Phys. Chem. B* **1999**, *103*, 6653 [(e)] Measured in this work *vs* Ag/AgCl and converted to *vs* NHE for consistency. [(f)] Estimated as described in section 7.1.

8.2.Brown's σ^+ constants vs E^0_{red} correlations

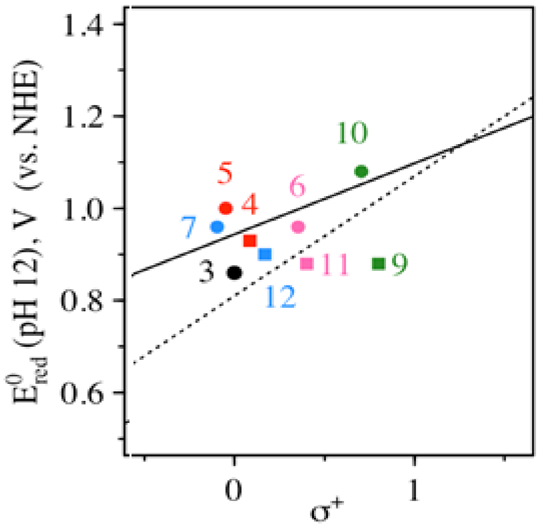

Figure S6. A plot of E^0_{red} (pH 12) as a function of Brown's σ^+ constants for chlorosubstituted phenols as reported by Hoffmann *et al.*[S15] and experimentally determined E^0_{red} for fluorophenols. The dashed line corresponds to the linear correlation E^0_{red} = 0.81+0.26 reported by Hoffmann *et al.*[S15] and solid line shows the correlation obtained for the fluorosubstituted phenols. Fluoro and chloro phenols are represented by spheres and squares, respectively. 3 = H, 4 = 2-Cl, 5 = 2-F, 6 = 3-F, 7 = 2,6-F, 9 = 2,5-Cl, 10 = 3,5-F, 11 = 3-Cl, 12 = 2,6-Cl.

Fig S6 shows a correlation plot of Brown σ^+ constants *vs.* E^0_{red} of chloro and fluoro substituted phenols at pH 12. A linear regression analysis of data points for fluorophenols (solid line) yields a slope of 0.15 and same analysis for chlorophenols (dashed line) yields slope of 0.26. The correlation plot reveals a general trend of higher reduction potentials of fluorophenols as compared to the cholophenols. This analysis is in line with high electronegativity of fluoro substituents than that of chloro.

The plot (Fig S6) was added as an independent estimate of the E^0 value for compound **7**. The spread in calculated rate constants in fig 1 for compound **7** is the difference between the electrochemistry data and that estimated from σ^+ correlations in fig S6. As the electrochemically derived value of E^0 is close to the correlation line for fluorophenols the spread in calculated rate constant values for **7** is small.

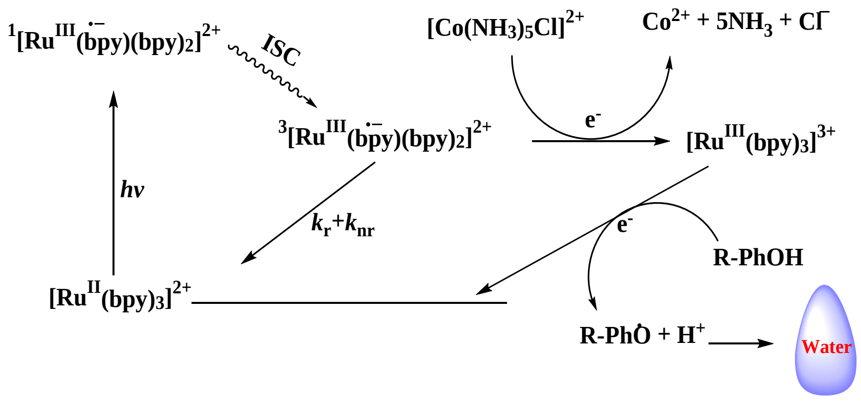

Scheme S1. General scheme for the oxidation of phenols by oxidized Ru(III) metal center in neat water at low pH (experimental conditions) and room temperature. ISC = intersystem crossing, k_r = rate constant for radiative decay, k_{nr} = rate constant for non-radiative decay.

9. References

S1. S. D. Glover, C. Jorge, L. Liang, K. G. Valentine, L. Hammarström and C. Tommos, *J. Am. Chem. Soc.*, 2014, 136, 14039-14051.

S2. D. V. Bent and E. Hayon, *J. Am. Chem. Soc.*, 1975, 97, 2599-2606.

S3. J. Feitelson and E. Hayon, *J. Phys. Chem.*, 1973, 77, 10-15.

S4. Y. Kajii, M. Fujita, H. Hiratsuka, K. Obi, Y. Mori and I. Tanaka, *J. Phys. Chem.*, 1987, 91, 2791-2794.

S5. G. N. R. Tripathi and R. H. Schuler, *J. Chem. Phys.*, 1984, 81, 113-121.

S6. H. Shindo and J. Hiraishi, *Chem. Phys. Lett.*, 1981, 80, 238-241.

S7. A. Juris, V. Balzani, F. Barigelletti, S. Campagna, P. Belser and A. von Zelewsky, *Coord. Chem. Rev.*, 1988, 84, 85-277.

S8. M. Sjödin, T. Irebo, J. E. Utas, J. Lind, G. Merényi, B. Åkermark and L. Hammarström, *J. Am. Chem. Soc.*, 2006, 128, 13076-13083.

S9. M. Gutman, in *Methods Biochem. Anal.*, John Wiley & Sons, Inc., 2006, DOI: 10.1002/9780470110515.ch1, pp. 1-103.

S10. R. A. Marcus and N. Sutin, *Biochim. et Biophys. Acta (BBA) - Reviews on Bioenergetics*, 1985, 811, 265-322.

S11. S. Hammes-Schiffer, *Acc. Chem. Res.*, 2001, 34, 273-281.

S12. C. P. Andrieux, J. Nadjo and J. M. Saveant, *J. Electroanal. Chem. Interfacial Electrochem.*, 1973, 42, 242.

S13. J. Stradins and B. Hasanli, *J. Electroanal. Chem.*, 1993, 353, 57-69.

S14. J. Lind, X. Shen, T. E. Eriksen and G. Merenyi, *J. Am. Chem. Soc.*, 1990, 112, 479-482.

S15. C. Li and M. Z. Hoffman, *J. Phys. Chem. B*, 1999, 103, 6653-6656.
